# DNA Modified with Boron–Metal Cluster Complexes [M(C_2_B_9_H_11_)_2_]—Synthesis, Properties, and Applications

**DOI:** 10.3390/ijms19113501

**Published:** 2018-11-07

**Authors:** Agnieszka B. Olejniczak, Barbara Nawrot, Zbigniew J. Leśnikowski

**Affiliations:** 1 Screening Laboratory, Institute of Medical Biology, Polish Academy of Sciences, 106 Lodowa St., 93-232 Lodz, Poland; aolejniczak@ibmpan.pl; 2 Centre of Molecular and Macromolecular Studies, Polish Academy of Sciences, Sienkiewicza 112, 90-363 Lodz, Poland; bnawrot@cbmm.lodz.pl; 3 Laboratory of Molecular Virology and Biological Chemistry; Institute of Medical Biology, Polish Academy of Sciences, 106 Lodowa St., 93-232 Lodz, Poland

**Keywords:** DNA, metallacarboranes, boron clusters, oligonucleotide probes, therapeutic nucleic acids

## Abstract

Together with tremendous progress in biotechnology, nucleic acids, while retaining their status as “molecules of life”, are becoming “molecular wires”, materials for the construction of molecular structures at the junction between the biological and abiotic worlds. Herein, we present an overview of the approaches for incorporating metal centers into nucleic acids based on metal–boron cluster complexes (metallacarboranes) as the metal carriers. The methods are modular and versatile, allowing practical access to innovative metal-containing DNA for various applications, such as nucleic acid therapeutics, electrochemical biosensors, infrared-sensitive probes, and building blocks for nanoconstruction.

## 1. Introduction

Metal complexes, due to their versatility in redox and photophysical features, together with well-defined, though diverse coordination geometries, represent a class of advantageous functional components with broad applicability for nucleic acids modification. The incorporation of metal centers into nucleic acids offers difficult to compete opportunities to create a new type of material that merges the informational properties of nucleic with the electronic, magnetic, and optical properties characteristic of metals and their complexes. As a result, materials possessing physical and chemical features that differ significantly from those accessible in bio-organic or inorganic compounds only can be fabricated [1,2].

One can distinguish between two different goals of nucleic acid modification. The first focuses on modulation and improvement of the natural properties of nucleic acids such as specificity and selectivity of binding towards complementary sequences or stability in a biological environment in order to improve the designed, specific activities. This approach is mainly used if modified nucleic acids are applied as therapeutic agents such as antisense oligonucleotides, ribozymes, or interfering RNAs, and perform their natural or nearly natural functions in biological systems [3]. 

The second goal of nucleic acids modification focuses on incorporation of new, “unnatural” properties into their structures such as fluorescence, luminescence, specific redox activity, radioactivity, and specific affinity-bearing residues. The purpose of these modifications is to equip a nucleic acid with constituents that provide detectable signals under designed conditions. In these technological rather than biological applications, a modified nucleic acid is often used as a material for the construction of diagnostic tests and biosensors. Modified DNA-oligomers used as primers for polymerase chain reaction (PCR) also find broad and diverse applications in molecular biology and molecular medical diagnostics. New applications of nucleic acids as materials for nanoconstruction are still emerging [4,5]. 

There is a wide variety of entities designed for nucleic acids modification, including the following: (1) fluorescent and affinity labels, (2) redox labels, and (3) modified nucleobases with different properties and applications. There are also different types of metal complexes and organometallic compounds used for that purpose. Some of them have been employed in molecular medicine, diagnostics, and technology, providing a range of useful advantages and practical applications [6,7,8]. The metal-containing nucleic acids can be divided into the following types: (1) nucleic acids with a metal incorporated in the form of a complex with chelate, (2) metallated-nucleic acids containing metal ions that form a complex directly with double-stranded DNA, and (3) metal-labeled nucleic acids containing metal complexes covalently bound to the DNA strand. The latter type is a major focus of the present outline. Detailed discussion of the specific topics of chemistry and applications of metal-containing nucleic acids is available in several general reviews and books published recently [4,5,9,10]. The present overview is focused on the modification of nucleic acids with metallacarboranes, a unique class of metal complexes consisting of metal ion(s) and boron clusters as metal ligands.

## 2. Metallacarboranes

**Figure 1:**
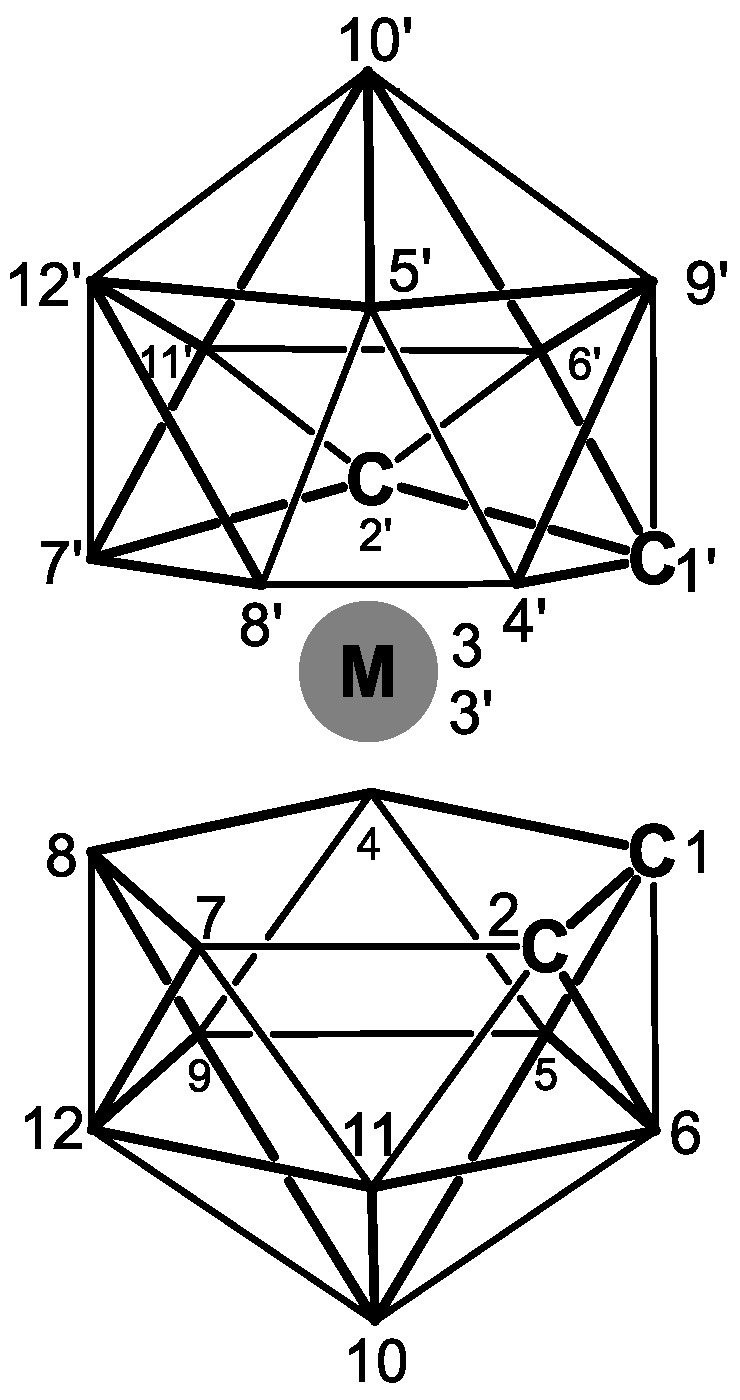
Structure and atom numbering of 3-metal-bis(1,2-dicarbollide) ate [M(C_2_B_9_H_11_)_2_]^−^. M = Co, Fe, Ni, Cr, Re, Al, Au, Ir, Mn, Pt, etc. Hydrogen atoms of C–H groups and B–H groups of the boron clusters, symbolized by the polygen vertex, were omitted for the clarity.

Polyhedral boron-rich compounds (boron clusters: *closo*-borates and carboranes) [11] have a broad spectrum of practical applications, from catalysis, optoelectronics, and new materials to medical chemistry and drug design [12,13]. One of the important features of a boron-rich system, such as the dicarbaborate anion (*nido*-7,8-C_2_B_9_H_12_^2−^), is the ability to serve as a ligand to form stable complexes with metal ions, that is, metallacarboranes [14]. Metallacarboranes are a large family of metallocene-type complexes containing at least one carborane polyhedron and one or more metallocenters (M = Co, Fe, Ni, Cr, Re, Al, Au, Ir, Mn, Pt, etc.) (Figure 1).

Metallacarboranes can be viewed as metal sandwich complexes in which a metal ion is facially coordinated to a carborane ligand analogous to metal–cyclopentadiene interactions in metallocenes. In most cases, the metal–carborane binding is strong, and separation of the ligand from the metal is rarely observed. The electron-delocalized bonding in these compounds has a strong stabilizing influence. Thus, both high and low formal metal oxidation states are stabilized as the effects of oxidation or reduction at the metal center are moderated via distribution over the cage skeleton [15].

Two general approaches have been developed for the modification of nucleic acids with carboranes and metallacarboranes: (1) de novo, step-by-step chemical synthesis of DNA oligomers with the use of metallacarborane-modified nucleoside monomers [16] for incorporation of the metallacarborane at specific locations; and (2) post-synthetic attachment of the metallacarborane to the suitably functionalized DNA oligonucleotides [17,18]. Recently, an enzymatic method for DNA modification with carboranes was also developed [19]. The expansion of the enzymatic approach for the incorporation of metallacarboranes into the nucleic acid chain seems feasible.

**Scheme 1:**
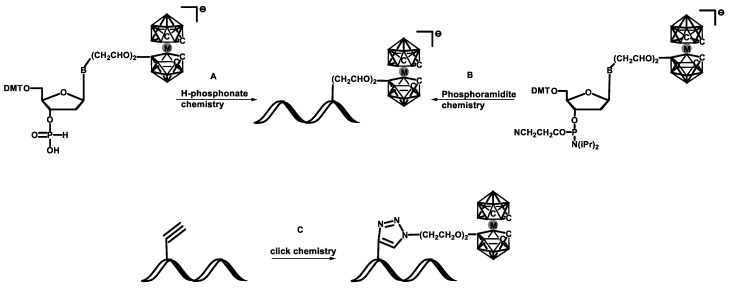
Methods for the synthesis of oligonucleotides modified with metallacarborane: (A) de novo solid-phase, step-by-step synthesis employing H-phosphonate chemistry; (B) de novo solid-phase, step-by-step synthesis employing phosphoramidite chemistry; (C) post-synthetic one-step procedure based on copper(I)-catalyzed Huisgen–Meldal–Sharpless 1,3-dipolar cycloaddition of azides and alkynes. Hydrogen atoms of C–H groups and B–H groups of the boron clusters, symbolized by the polygen vertex, were omitted for clarity.

DNA containing metallacarborane groups via de novo synthesis was obtained using the following methods: (A) solid-phase synthesis employing H-phosphonate chemistry or (B) solid-phase synthesis employing phosphoramidite chemistry. The post-synthetic modification approach (C) is based on a one-step procedure employing copper(I)-catalyzed Huisgen–Meldal–Sharpless 1,3-dipolar cycloaddition of azides and alkynes [20] (Scheme 1).

## 3. Synthesis of DNA Modified with Metallacarborane Complexes via de novo, Step-by-Step Chemical Synthesis

**Scheme 2:**
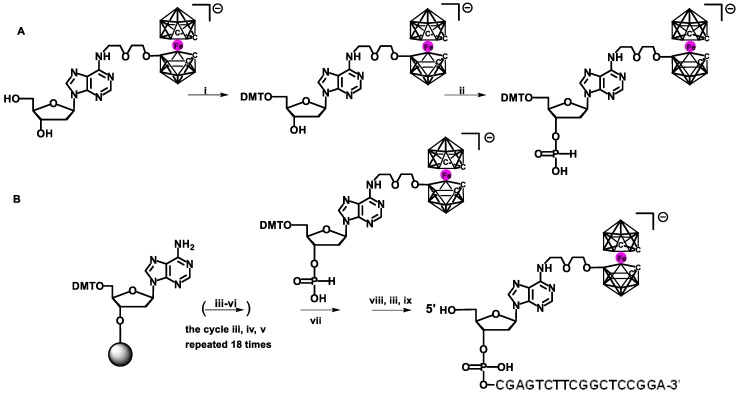
Solid-phase synthesis of DNA modified with metallacarborane using H-phosphonate chemistry. (**A**) Synthesis of the H-phosphonate monomer: (i) dimethoxytrityl chloride (DMTCl)l and pyridine; (ii) PCl_3_, imidazole, Et_3_N, CH_2_CH_2_, and H_2_O, −40 °C. (**B**) Synthesis of DNA oligonucleotides: (iii) detritylation: 3% dichloroacetic acid (DCA) in CH_2_Cl_2_; (iv) coupling: unmodified monomers, phosphoramidite chemistry; (v) oxidation: 0.1 mol/L I_2_ in THF/pyridine/H_2_O (13:6:1); (vi) capping: 1 mol/L (CH_3_CO)_2_O in THF/pyridine (1:8) and 0.5 mol/L 4-dimethylaminopyridine (DMAP) in THF; (vii) coupling: modified monomer, H-phosphonate chemistry; (viii) oxidation: 10% H_2_O in CCl_4_, Et_3_N, *N*-methylimidazole (9.0:0.5:0.5, *v*/*v*/*v*); (ix) cleavage from the support: 30% NH_3_ aq., 1 h, RT; base deprotection: 30% NH_3_ aq. 2 h, 50 °C. Hydrogen atoms of C–H groups and B–H groups of the boron clusters, symbolized by the polygen vertex, were omitted for clarity.

Originally, H-phosphonate chemistry was applied to synthesize oligonucleotides modified with carborane and metallacarborane of a sequence complementary to the fragment of the UL55 gene of human cytomegalovirus (HCMV) [21]. The solid-phase synthesis of modified oligonucleotide containing the 3,3′-iron-1,2,1′,2′-dicarbollide (BEFeC) at the 5′ end of 5′-d(^BEFeC^ACGAGTCTTCGGCTCCGGA)-3′ oligomer was performed using an automated DNA synthesizer. The β-cyanoethyl phosphoramidite chemistry was used to synthesize the unmodified part of the oligonucleotide, and H-phosphonate chemistry was used to add the modified monomer bearing the metallacarborane moiety (Scheme 2). The expected nucleoside composition and the presence of the metallacarborane modification in the obtained oligomer was confirmed by enzymatic digestion [21]. Metallacarborane-containing dinucleotides bearing metallacarborane complexes were also synthesized using H-phosphonate chemistry and the solution-phase synthesis mode [22]. The same oligonucleotide, 5′-d(^BEFeC^ACGAGTCTTCGGCTCCGGA)-3′, has been used as a target for biosensor devices [23]. The aqueous solution of the modified oligonucleotide was placed onto the electrode to allow hybridization with the unmodified oligonucleotide. Electrochemical signals were observed from the metallacarborane group, especially when a 3,3′-iron-1,2-1′,2′-dicarbollide complex was located far away from the electrode after hybridization. These studies confirmed the usefulness of the metallacarborane-containing Fe (III) ion as an effective redox marker.

**Scheme 3:**
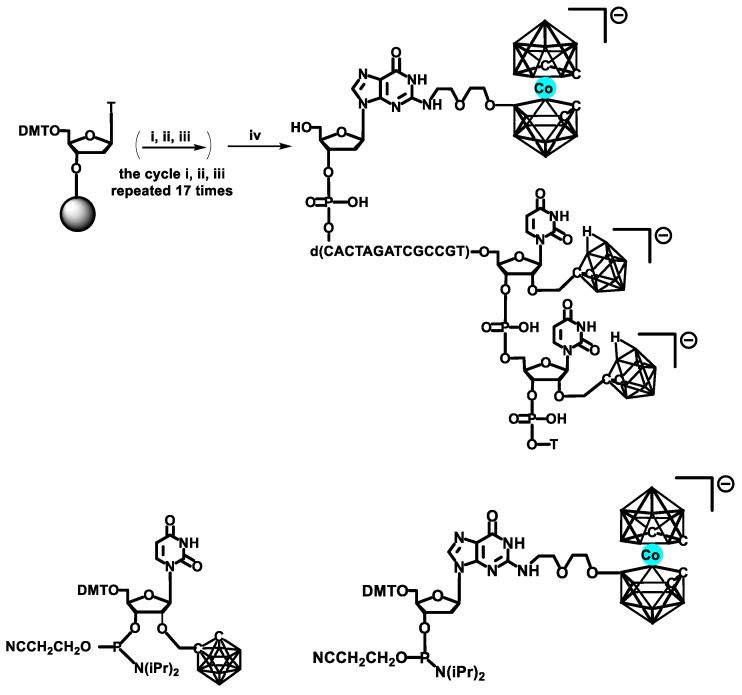
Solid-phase synthesis of DNA modified with carborane and metallacarborane using phosphoramidite chemistry: (i) detritylation, (ii) coupling, (iii) oxidation and capping, and (iv) cleavage from the solid support and deprotection [25]. Hydrogen atoms of C–H groups and B–H groups of the boron clusters, symbolized by the polygen vertex, were omitted for clarity.

DNA oligomers modified simultaneously with carborane (2′-*O*-(*o*-carboran-1-yl)methyluridine, 2′-CBM) and metallacarborane (2-*N*-{5-[3-cobalt bis(1,2-dicarbollide)-8-yl]-3-oxa-pentoxy}-2′-*O*-deoxyguanosine, BEMC) nucleosides complementary to the insulin receptor substrate (IRS-1) gene were also synthesized using a phosphoramidite approach for the introduction of carborane or metallacarborane groups-containing nucleoside monomers to the oligonucleotide [24]. Unmodified parts of the oligonucleotides were prepared by automated solid-phase synthesis (Scheme 3). The manual step was used for the insertion of phosphoramidites of 2′-CBM and BEMC bearing carborane or metallacarborane modification, respectively. It should be noted that because of the treatment of the oligonucleotide product with a concentrated solution of ammonia in water during the cleavage from the solid support and deprotection, the electroneutral *closo*-form of the carborane in 2′-CBM is transformed into a negatively charged *nido*-counterpart.

The physicochemical and biological properties of modified DNA were studied. The lipophilicity and resistance to enzymatic degradation by snake venom phosphodiesterase (SVPDE) of the modified oligonucleotides was higher than the unmodified counterparts, especially in the case of simultaneous modification at the 3′ and 5′ ends with 2′-CBM and BEMC groups, respectively. An effective formation of the doubled stranded structure with the complementary DNA-oligomers was shown [24].

5′-*O*-Monomethoxytrityl-4-*O*-diethyleneoxy-8-[3-cobalt bis(1,2-dicarbollide)-8-yl]-thymidine- 3′-O-(*N*,*N*-diisopropyl-β-cyanoethyl)phosphoramidite was used to synthesize DNA containing metallacarborane, 5′-d(^BEMC^TGCTGGTTTGGCTG)-3′, using a β-cyanoethyl phosphoramidite cycle and an automated DNA synthesizer. The metallacarborane-modified nucleoside was attached to the 5′ end of the oligomer. In the oxidation step, *t*-BuOOH was applied instead of I_2_, which is routinely used as the oxidizing agent. The synthesized oligonucleotide labeled with a metallacarborane cage was used as one of two primers in polymerase chain reaction (PCR) amplification of a fragment of HCMV DNA, strain AD 169. The other primer was unmodified oligonucleotide P12 (5′-AAACGGCGCAGCCACATAAGG-3′). The susceptibility of the primer with metallacarborane modification at its 5′ end to an extension by Taq DNA polymerase was proved by PCR amplification of the HCMV genome fragment on the junction of US14 and US13 gene. The 812-bp amplification product was obtained as expected [26].

**Scheme 4:**
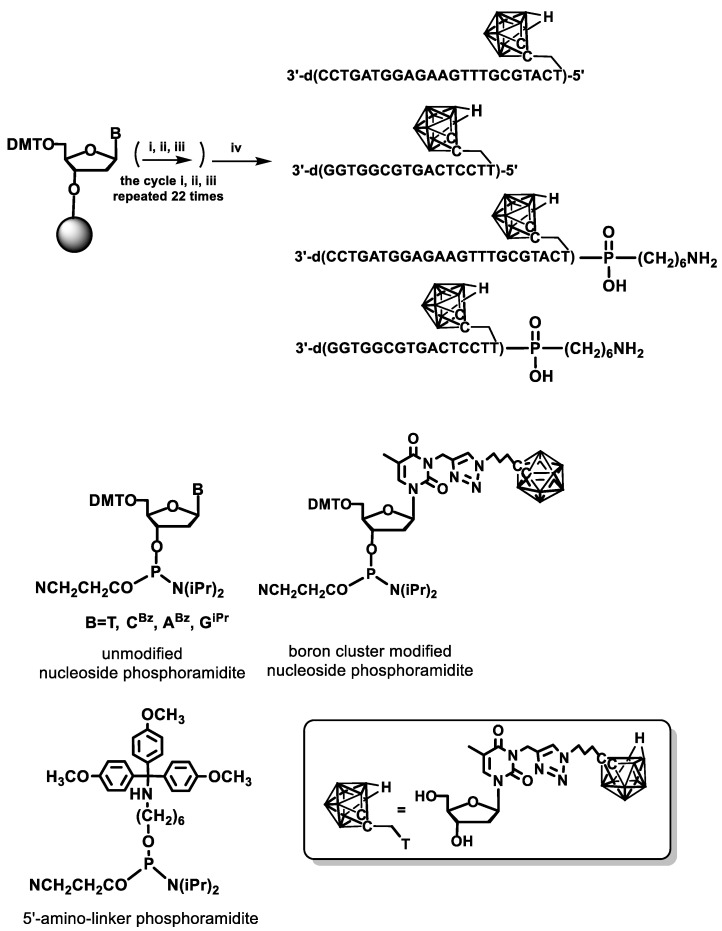
Synthesis of oligonucleotides containing carborane cluster at the 5′-end of the oligonucleotide chain: (i) detritylation; (ii) coupling; (iii) oxidation and capping; (iv) cleavage from the solid support and deprotection. Hydrogen atoms of C–H groups and B–H groups of the boron clusters, symbolized by the polygen vertex, were omitted for clarity.

Expanding these works, we have developed a general method for the synthesis of four canonical nucleosides T, dC, dA, and dG modified with 1,2-dicarba-*closo*-dodecaborane (C_2_B_10_H_11_) cage, and their phosphoramidites, suitable for automated synthesis of DNA [27]. The modified nucleoside monomers bearing boron cluster were prepared from suitable 5-ethynyl derivatives in one-step reaction using cooper(I)-catalyzed Huisgen–Meldal–Sharpless 1,3-dipolar cycloaddition of azides and alkynes to give triazoles. The 5′-*O*-(4,4′-dimethoxytrityl)-3′-*O*-(*N*,*N*-diisopropyl-β-cyanoethyl)-*N*^3^-{[(*O*-carboran-1-yl)propyl)]-1*N*-1,2,3-triazol-4-yl}methylenethymidine phosphoramidite was used to synthesize four modified DNA oligonucleotides of various lengths and sequences of nucleobases. All the oligomers contained one carborane modification at the 5′-end, and two of them were additionally equipped with an amino linker C6 (Scheme 4). The oligonucleotides were obtained by solid-phase synthesis using a standard β-cyanoethyl cycle [25].

**Scheme 5:**
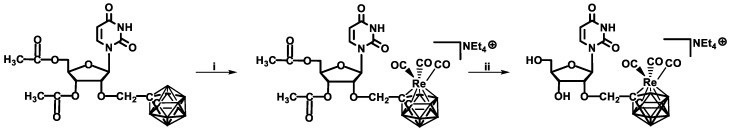
Synthesis of a uridine-bearing 3,3,3-(CO)_3_-*closo*-3,1,2-ReC_2_B_9_H_10_ complex: (i) TEAF_aq_., EtOH, [Net_4_]_2_[ReBr_3_(CO)_3_], 100 °C, 30 h; (ii) 25% NH_3aq._, MeOH, 35 °C, 30 min. Hydrogen atoms of C–H groups and B–H groups of the boron clusters, symbolized by the polygen vertex, were omitted for clarity.

Mass spectrometry analysis proved that the carborane cage is, as expected, in *nido*-form (7,8-dicarba-*nido*-undecaborate ion). The conversion of the boron cluster from *closo*- to *nido*-form takes place during the cleavage of the synthesized oligomer from the support and nucleic bases’ deblocking under treatment with concentrated ammonia solution in water [28]. The modified oligonucleotides containing negatively charged, redox active 7,8-dicarba-*nido*-undecaborate have been used as a probe for electrochemical detection of specific DNA sequences derived from the cytomegalovirus (HCMV) [29]. Alternatively, they can be used as precursors for post-synthetic modification of the oligonucleotides with metallacarborane unit based on the 3,3,3-tricarbonyl-3-metal complex formation with 7,8-dicarba-*closo*-dodecaboran-1-yl ligand [30], according to the procedure shown in Scheme 5.

Using this approach, synthesis of a new type of nucleoside–metallacarborane conjugate, 2′-*O*-(3,3,3-tricarbonyl-3-rhenium-7,8-dicarba-*closo*-dodecaboran-1-yl) methyluridine, was recently achieved [31]. The method was based on the de novo formation of a metallacarborane complex via the reaction of [Net_4_]_2_[ReBr_3_(CO)_3_] with the uridine-bearing carborane as a metal ligand (Scheme 5). 

## 4. Post-Synthetic Labeling of DNA with Metallacarboranes via a “Click Chemistry” Approach

**Scheme 6:**
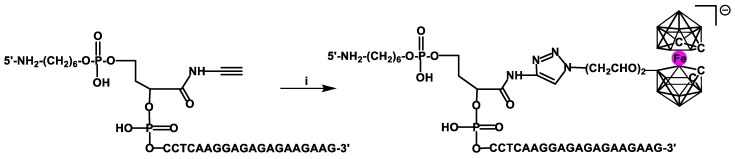
Synthesis of DNA containing a metallacarborane moiety using Huisgen–Meldal–Sharpless cycloaddition (HMSC): (i) tris[(1-benzyl-1*H*-1,2,3-triazol-4-yl)methyl]amine (TBTA), sodium ascorbate, CuSO_4_ × 5 H_2_O, [8-N_3_-(CH_2_CH_2_O)_2_-3-iron bis(dicarbollide)], H_2_O. Hydrogen atoms of C–H groups and B–H groups of the boron clusters, symbolized by the polygen vertex were omitted for clarity.

The CuI-catalyzed 1,3-dipolar Huisgen–Meldal–Sharpless cycloaddition (HMSC) of azide and alkyne leads to the formation of a triazole moiety. This process, often called “click chemistry” or “chemical ligation”, became an important tool for chemical modification of biomolecules [20,32]. The azide and alkyne components have several advantages. They can be conveniently introduced in an independent manner, are relatively stable and do not react with common organic reagents nor with a majority of functional groups present in biomolecules (are orthogonal). The formation of triazoles is irreversible and usually proceeds in high yield. In addition, this reaction is remarkably versatile because of the extremely mild and chemoselective copper(I) catalyst system, which is surprisingly insensitive to many solvents and operate well in a wide pH range. The advantages of the “click chemistry” approach, based not only on HMSC but also on other types of bioorthogonal chemistries such as Diels–Alder “click” reactions, allow for its application in areas such as synthesis on solid supports, combinatorial chemistry, or “organic chemistry in vivo” [33]. The click chemistry approach was used to label a DNA oligomer with the 3,3′-iron-1,2,1′,2′-dicarbollide group [34,35] using an alkyne derivative of DNA (with an acetylene moiety located close to the 5′ end) and an azide component bearing the metallacarborane group at the end of a diethylenoxy linker (Scheme 6).

The obtained oligonucleotide deposited on a gold electrode acted as a main part of a novel electrochemical genosensor capable of electrochemical determination of the DNA sequences derived from avian influenza virus (AIV), type H5N1. The oligonucleotide probes containing an NH_2_ group close to the 5′-end nucleotide were covalently attached to the electrode via coupling of *N*-hydroxysuccinimide/N-(3-dimethyl-aminopropyl)-*N*-ethylcarbodiimide hydrochloride (NHS/EDC) and 3-mercaptopropionic acid. The latter was previously deposited on the surface of gold electrode as a self-assembled monolayer (SAM). For the measurement, an Osteryoung square-wave voltammetry method was applied. The changes in the redox activity of the Fe(III) center of the metallacarborane complex before and after the hybridization process were found to be diagnostic and displayed good selectivity and sensitivity towards several complementary targets derived from AIV, H5N1. The calculated detection limits recorded for an ssDNA 20-mer and PCR products having complementary sequences at the 3′ end and in the middle of the oligonucleotide based on experimental data ranged from 0.03 to 0.08 fM, respectively; thus the genosensor was superior to many others previously reported. Its additional advantage was the ability to distinguish PCR products containing complementary sequences in different positions [34].

**Scheme 7:**
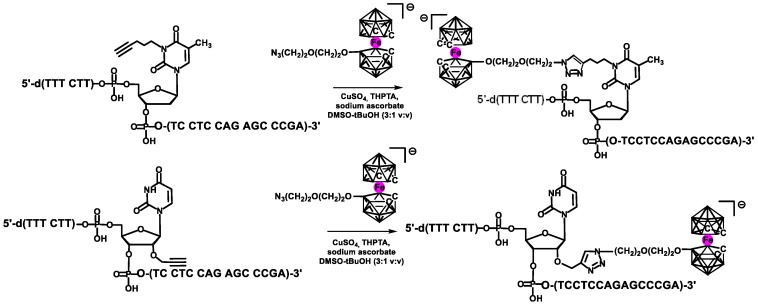
Example synthesis of boron cluster-modified anti-epidermal growth factor receptor (EGFR) antisense oligonucleotides via post-synthetic modification using HMSC. DNA oligonucleotides bearing 3-*N*-metallacarborane-modified thymidine unit(s) (as in the upper reaction scheme) are assigned thymidine (TB)-type, while those bearing 2′-*O*-metallacarborane modified uridine units (a lower panel) are assigned uridine (UB)-type. Adapted in part from the literature [35]. Hydrogen atoms of C–H groups and B–H groups of the boron clusters, symbolized by the polygen vertex, were omitted for clarity.

**Scheme 8:**
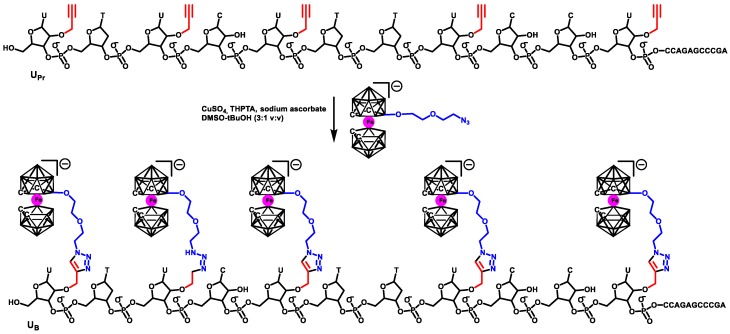
Antisense UB-type DNA oligonucleotide targeted EGFR obtained via Cu(I)-assisted “click” conjugation, bearing five Fe (III)-metallacarborane units. Hydrogen atoms of C–H groups and B–H groups of the boron clusters, symbolized by the polygen vertex, were omitted for clarity.

Another type of HMSC driven labeling of nucleic acids with metallacarboranes (shown in Scheme 7 and Scheme 8) consisted of two steps [35]. First, oligonucleotides containing, at a given position, a thymidine unit modified with 4-pentyn-1-yl substituent at the position 3-N were obtained by the use of corresponding phosphoramidite monomer. Also, oligomers bearing a uridine unit modified with a 2′-*O*-propargyl group were synthesized using commercially available monomer. In the second step, the alkyne-modified oligomers were post-synthetically conjugated to alkyl azide derivatives of boron clusters via HMSC [20,36]. 

For the proof of concept, a 22-mer antisense oligodeoxyribonucleotide (ASO) of the sequence 5′-d(TTT CTT TTC CTC CAG AGC CCGA)-3′ was chosen, which recognizes the epidermal growth factor receptor (EGFR) mRNA, known for being overexpressed in cancerous cells. The synthesized ASO oligonucleotides contained one or two thymidine or uridine metallacarboranes conjugates (of thymidine (TB)-type or uridine (UB)-type, respectively; Scheme 7) in positions 7 or/and 11 of the oligomer chain [35]. The metallacarborane moieties were anchored at position 3-*N* of thymidine (TB) or alternatively at 2′-oxygen atom of uridine (UB) unit. In addition, two UB-type ASOs (heavily loaded with metallacarborane, Scheme 8) [37] were also obtained according to the above procedure (see Table 1 for the sequences and modification type and position). The obtained models were used for evaluation of physicochemical and biological properties of DNA–metallacarborane conjugates.

## 5. Physicochemical and Biological Properties of Metallacarborane Modified Oligonucleotides of TB- and UB-Types

*Binding affinity to complementary sequences.* The affinity of the metallacarborane modified oligomers of TB- or UB-type (Table 1) to their complementary target RNA strand 3′-AAA GAA AAG GAG GUC UCG GGCU-5′ was assessed by the thermal dissociation assay. The measurement of melting temperatures (Tm) has shown that metallacarborane modified ASO/RNA duplexes are thermodynamically less stable than the reference duplex containing the non-modified ASO strand (NM-ASO) complexed with complementary RNA (NM-ASO/RNA). However, the presence of the UB-type modifications decreased the duplex stability less than those of the TB-type. This is because the nucleotide of UB-type with the metallacarborane moiety present at the 2′-position adopts preferentially the C3′-*endo* conformation typical for the RNA units, and thus it well accommodates in the DNA/RNA helices, known for adopting the overall conformation of the A-type. 

In contrast, in the TB unit, the metallacarborane group occupies the 3-*N* position of nucleobase, therefore, there is a disruption of the Watson–Crick base pairing with the complementary adenine nucleotide. Thus, one may conclude that ASOs modified with the UB-type units are advantageous for recognition of the target mRNA sequences and activation of the RNase H, a prerequisite for gene expression regulation by the antisense approach [38].

*Lipophilicity of UB- and TB-type ASOs*. The metallacarborane units lead to substantially increased lipophilicity of their oligonucleotide conjugates (listed in Table 1) compared with the non-modified reference precursor MN-ASO, as shown by the values of the partition coefficients in the water/1-octanol bi-phase system (Log*P*) [35] and by the changes of chromatographic mobility on a C18 reverse phase HPLC column (R_T_ are given in parentheses in Table 1) [35,37]. The increasing number of the UB units loaded leads to the linear increase of the lipophilicity of the UB-type ASOs [35,37].

*Circular dichroism (CD) measurements of UB-type ASOs*. CD is a powerful tool for monitoring structural changes of DNA and RNA duplexes resulting from the presence of modified units in their strands. As shown previously, the CD spectra of the UB-type ASO bound to the complementary RNA strand exhibit only minute deviations from the CD profile of the reference, non-modified DNA/RNA duplex [35,37]. This observation indicates that the UB-type units inserted in the oligonucleotide chain slightly alter the structure of the ASO/RNA duplexes. This feature is important for recognition of mRNA and for induction of the RNase H activity. In this respect, the UB-type ASO metallacarborane conjugates may be useful in the antisense or boron neutron capture therapy (BNCT) applications. 

*Infrared (IR) activity of metallacarborane labeled DNA*. To date, detection of nucleic acids using infrared light sensitive labels and IR spectroscopy has not been used very often, mostly because of the lack of labels absorbing electromagnetic radiation within a 1900–2600 cm^−1^ window, where nucleic acids (and typical organic molecules) are transparent. Boron clusters attached to DNA or RNA oligonucleotides make a difference because IR spectra of carborane (C_2_B_10_H_12_) or metallacarborane [3-Co-(1,2-C_2_B_9_H11)_2_]^−^ moieties contain a unique band attributed to the B–H vibration at ca. 2600 cm^−1^ [39,40]. The HMSC method allowed for further improvement of this labeling approach. Incrusting of an oligonucleotide probe with multiple metallacarborane labels, each containing 18 B–H bonds, resulted in significant enhancement of the diagnostic B–H signal and increased sensitivity of the detection [37]. For ASOs of the UB-type, containing four or five metallacarborane units at positions 2, 5, 8, 11 or 1, 3, 5, 8, 11 of the oligonucleotide chain, the B–H related bands occurred in the 2500–2650 cm^−1^ region, similar to the signals in free Fe(III)-metallacarborane [3-Fe-(1,2-C_2_B_9_H11)_2_]^−^, as well as the signals of the B–H bonds found in uridine nucleoside bearing Fe(III)-metallacarborane modification at 2′-position [37]. Thus, the metallacarborane moieties inserted into the oligonucleotide chain show the bands characteristic for the B–H vibration and can be used as an infrared light sensitive marker.

*Biological evaluation of the UB-type ASOs*. The silencing activity (inhibition of protein biosynthesis) of boron cluster-modified ASOs was tested against the mRNA of EGFR protein in a dual florescence assay (DFA), in which the fusion plasmid coding green fluorescent protein (GFP) and the target fragment of EGFR (pEGFP-EGFR) was exogenously expressed in HeLa cells, and was assessed by comparison of the level of fluorescence of EGFP and of the control red fluorescent protein (RFP) simultaneously expressed from pDsRed-N1 plasmid. Considerable silencing activity, depending upon the location of the modification within the oligonucleotide chain, was observed for the selected ASOs. 

In the antisense approach, the ASO oligonucleotides operate by sequence-specific hybridization to the complementary RNA followed by recruitment of RNase H, which hydrolyses the target RNA. Although the requirements that the ASOs have to meet to inhibit a protein expression are not known precisely, modifications in a sugar moiety, for example, change of the sugar type or its orientation, are reported to affect the RNase H activation [41]. From the other side, borane clusters are known for binding to the cellular proteins [42] and modulation of their cellular function. Therefore, the observed differences in the silencing activity of the ASO-metallacarborane conjugates may be of different origin. In the reported case, single modified ASOs of the TB- and UB-type in position 11 of the ASO chain (as in TB_11_ and UB_11_) used in 200 nM concentration exhibit enhanced silencing potential compared with the reference ASO (by ca. 20%, Table 1). In contrast, location of the metallacarborane units at position 7 of ASOs (as in TB_7_ and UB_7_) decreased their silencing activity. For highly modified UB-type ASOs, the silencing effect is balanced by positive and negative features of the metallacarborane units, leading to ASOs of similar antisense activity as the reference ASO.

*Redox properties ofFe (III)-metallacarborane ASO conjugates*. The tested ASOs, when used at low concentrations (up to 50 μM), exhibited pro-oxidative activity by inducing reactive oxygen species (ROS) production and an increase in the mitochondrial activity of HeLa cells. In contrast, when used at higher concentrations, the ASOs exhibited antioxidative properties, lowering the level of ROS [37].

Inside cells, the highly metallacarborane-loaded ASOs containing redox-active Fe(III) ions generate ROS as a result of oxidative stress. At low concentrations, these compounds enhance the metabolic activity. Along with the increase of concentration, the amount of Fe(III) ions (free radicals scavenger) also increases, so the antioxidative properties of the ASOs are observed. When concentration of ASOs reaches ≈200 nM, the ROS levels are the same as in the control cells, suggesting that the rates of ROS formation and elimination are equal. It is suggested that the silencing of EGFR by the use of high concentrations of oligonucleotide–metallocarborane conjugates occurs without noticeable increase of ROS concentration. It should be emphasized that the delivery of unmodified antisense and nonsense oligonucleotides did not result in a significant increase of the ROS levels. Therefore, it is hypothesized that the high metallacarborane-loaded DNA oligomers may be used as potential BNCT and antisense oligonucleotide (dual-action) anticancer agents.

## 6. Summary and Conclusions

DNA modified with boron cluster-metal complexes, metallacarboranes [M(C_2_B_9_H_11_)_2_], represent a new class of metal containing nucleic acids. Metallacarboranes provide probably the most versatile platform for modification of nucleic acids with metals, though this immense potential is awaiting full exploration. Not all metallacarboranes are stable in a biological (water) environment, but a plethora of metallacarboranes suitable for use as metal carriers for biomolecules’ modification can be selected. The methods described in this overview are modular and versatile, allowing practical access to innovative metal-containing DNA. These and new methods developed in future may open the way for more widespread use of metal-labeled nucleic acids for a variety of applications.

## Figures and Tables

**Table 1:** Binding affinity of the anti-epidermal growth factor receptor (EGFR) metallacarborane antisense oligonucleotides to the complementary RNA strand assessed by thermal dissociation assay (Tm), lipophilicity (Log*P*), RP-HPLC mobility (retention time R_T_), and antisense activity (antisense oligodeoxyribonucleotide (ASO) activity) assessed by a dual fluorescence assay (DFA) against EGFR mRNA.

Name	Oligonucleotide Sequence ^a^	Tm ^b^ [°C]	ASO Activity ^c^ [%]	Log*P* (R_T_^d^ [min])	Ref.
NM	5′-d(TTTCTTTTCCTCCAGAGCCCGA)-3′	69.5	65 ± 1	−1.17 ± 0.36 (11.6)	[35]
TB_7_	5′-d(TTTCTT**TB**_7_TCCTCCAGAGCCCGA)-3′	62.5	46 ± 4	−0.60 ± 0.15	[35]
TB_11_	5′-d(TTTCTTTTCC**TB**_11_CCAGAGCCCGA)-3′	57.5	82 ± 2	nd	[35]
TB_7,11_	5′-d(TTTCTT**TB**_7_TCC**TB**_11_CCAGAGCCCGA)-3′	45.0	24 ± 2	nd	[35]
UB_7_	5′-d(TTTCTT**UB**_7_TCCTCCAGAGCCCGA)-3′	65.5	34 ± 2	−0.71 ± 0.42 (17.5)	[35,37]
UB_11_	5′-d(TTTCTTTTCC**UB**_11_CCAGAGCCCGA)-3′	67.5	89 ± 2	nd	[35]
UB_7,11_	5′-d(TTTCTT**UB**_7_TCC**UB**_11_CCAGAGCCCGA)-3′	63.5	68 ± 1	nd (20.1)	[35,37]
UB_2,5,8,11_	5′-d(T**UB**_2_TC**UB**_5_TT**UB**_8_CC**UB**_11_CCAGAGCCCGA)-3′	60.9	54 ± 2	(22.2)	[37]
UB_1,3,5,8,11_	5′-d(**UB**_1_T**UB**_3_C**UB**_5_TT**UB**_8_CC**UB**_11_CCAGAGCCCGA)-3′	60.4	62 ± 3	(23.9)	[37]

^a^ The thymidine (TB) or uridine (UB) metallacarborane conjugates are inserted in the indicated positions of the oligonucleotide chains. ^b^ Melting temperatures (Tm) of ASO/RNA duplexes are given with ±0.5 °C accuracy. ^c^ Antisense activity of anti-EGFR ASO in HeLa cells at 200 nM concentration in DFA. ^d^ R_T_ [min]—RP HPLC retention time on a C18 reverse phase HPLC column, a gradient of acetonitrile in aqueous ammonium acetate solution (0.1 M CH_3_COONH_4_) from 0% to 70% of acetonitrile as an eluting solvent system was used.
